# Supplementary material for: Knowledge, attitudes and acceptance of COVID-19 vaccine among pregnant women in Mbeya Region
Source: PLOS Glob Public Health. 2025 Jul 24;5(7):e0004408. doi: 10.1371/journal.pgph.0004408 (PMC12288997; doi:10.1371/journal.pgph.0004408)
Supplement: S1 Text — (DOCX) [file pgph.0004408.s002.docx]

### Reliability

Reliability of the questionnaire on Knowledge (Qs), attitudes (qs) and acceptance towards COVID-19 vaccination(As) among pregnant women was tested by Cronbach's alpha which values were > 0.7 (showing internal consistency) as shown below .
