# Supplementary material for: Knowledge, attitudes and acceptance of COVID-19 vaccine among pregnant women in Mbeya Region
Source: PLOS Glob Public Health. 2025 Jul 24;5(7):e0004408. doi: 10.1371/journal.pgph.0004408 (PMC12288997; doi:10.1371/journal.pgph.0004408)
Supplement: S2 Text — (DOCX) [file pgph.0004408.s003.docx]

KNOWLEDGE ON COVID-19 VACCINE

Using Bloom's cut off point, respondents' overall knowledge was classed as good if their score was between 80 and 100%, moderate if their score was between 60 and 79%, and low if their score was less than 60%.

ATTITUDE TOWARDS COVID-19 VACCINE

Bloom's cut off point was used to categorize attitudes towards the COVID-19 vaccine as positive (80-100%), neutral (60-79%), or negative (< 60%).

COVID-19 VACCINE ACCEPTANCE

A specific item “If COVID-19 vaccine were recommended for pregnant women, would you get vaccinated?” those who responded “YES” for this question were considered as vaccine acceptance and those who responded “NO” for this question were regarded as vaccine hesitancy.

FACTORS ASSOCIATED WITH COVID-19 VACCINE ACCEPTANCE

Binary logistic regression and chi square statistics were run to identify factors associated with COVID-19 vaccine acceptance among pregnant women and a P value of < 0.05 was considered as “statistically significant”.

In binary logistic regression model output Education level(OR=0.35, CI 0.187-0.644, P=0.001), religion(OR=0.53, CI 0.317-0.887, P=0.016), number of people in the household(OR=0.49, CI 0.278-0.849, P=0.011), chronic medical illness(AOR=3.21, CI 1.448-7.123, P=0.004), high-risk pregnancies(OR=0.84, CI 0.455-1.553, P=0.006), reliable information sources(OR=2.53, CI 0.068-0.633,P=0.006), preference to natural immunity over vaccination(AOR=0.42, CI 0.341-0.498, P=0.018), trust in pharmaceutical companies ((OR=9.59, CI 4.992-18.431, P=0.000), attitudes toward the vaccine(AOR=1.26, CI 1.149-1.368, P=0.015), and COVID-19 vaccine knowledge(AOR=2.70, CI 2.587-2.810, P=0.005), prior vaccination history(AOR=0.13,CI 0.068-0.183, P=0.000), having being vaccinated against COVID-19 (AOR=0.67, CI 0.594-0.755, P=0.000) were determinants of COVID-19 vaccine acceptability (P-values <0.05)

A chi-square test was also used to investigate parameters linked with the observed Knowledge, Attitude and Acceptance for the COVID-19 vaccination. Fisher's exact test was used to identify significant variables, with a P-value <0.05 indicating statistical significance of the connections.

Poor knowledge of the COVID-19 vaccine was found to be connected with vaccine acceptance, with only 33.13% of pregnant women with low knowledge compared to 48.78% and 57.69% of those with moderate and good knowledge, respectively, having accepted the vaccine. (Chi=7.88, P**=**0.002).

Furthermore, only 6.02% of participants with poor knowledge of COVID-19 vaccine compared to 12.30% and 42.31% of those displayed moderate and good knowledge respectively had positive attitude towards COVID-19 vaccination (Chi**=**34.45, P=0.000).

Furthermore, negative attitude toward COVID-19 vaccination was a major determinant in vaccine acceptance, with only 34.08% of pregnant women with negative attitude, compared to 53.57% and 53.85% of those with neutral and positive attitude, respectively, accepting COVID-19 immunization. (Chi=6.74, P=0.034).
