# Supplementary material for: Knowledge, attitudes and acceptance of COVID-19 vaccine among pregnant women in Mbeya Region
Source: PLOS Glob Public Health. 2025 Jul 24;5(7):e0004408. doi: 10.1371/journal.pgph.0004408 (PMC12288997; doi:10.1371/journal.pgph.0004408)
Supplement: Attachment — Submitted filename: Response to Editor.docx [file pgph.0004408.s005.docx]

Comments from Editor

1. Please ensure that you refer to your Tables 6 and 7 in your text as, if accepted, production will need these references to link the reader to the tables.

- We have referred Tables 6 and 7 to ensure that these references link the reader to the tables.

2. Please provide separate main figure files in .tif or .eps format only and remove any figures embedded in your manuscript file. Please also ensure that all files are under our size limit of 10MB. Please leave the figure captions or legends in the manuscript.

For more information about how to convert your figure files please see our guidelines: https://journals.plos.org/globalpublichealth/s/figures

You may send us an email at globalpubhealth@plos.org if you are having difficulties converting figure files to the required format.

- We have provided separate main figure files in .tif format, removed embedded figures in the manuscript, ensured the files are less than 10M and left the figure captions in the manuscript.

3. At this time, there does not appear to be a minimal data set uploaded for this submission. PLOS journals require authors to make all data underlying the findings described in their manuscript fully available without restriction at the time of publication (https://journals.plos.org/globalpublichealth/s/data-availability) and not only summary statistics. However, we do allow for rare exceptions to this strict requirement in cases where there are ethical or legal considerations with respect to the public sharing of the underlying data.

Please also note that the data we ask authors to share is not necessarily the full and/or raw data set collected in the course of their research, but rather the "minimal data set", which we define as consisting of the data set used to reach the conclusions drawn in the manuscript with related metadata and methods, and any additional data required to replicate the reported study findings in their entirety. Depending on the nature of your study, this can include any or all of the following:

- The values behind the means, standard deviations and other measures reported;

- The values used to build graphs;

- The points extracted from images for analysis.

- We confirm that the minimal data set required to replace all findings in our manuscript will be made fully available upon publication. The complete minimal data set including all values behind reported statistics and analytical outputs.

Before we proceed with your manuscript, please address the following prompts:

a) If there are ethical or legal restrictions on sharing a de-identified data set, please explain them in detail (e.g., data contain potentially identifying or sensitive patient information) and who has imposed them (e.g., a Research Ethics Committee or Institutional Review Board, etc.). Please provide non-author contact information (phone/email/hyperlink) for a data access committee, ethics committee, or other institutional body to which data requests may be sent. If applicable, please also provide any necessary information which interested researchers would need when requesting access to data in order to obtain the minimal data set for your study.

- No legal /ethical restrictions on sharing the minimal set data applied.

b) If your minimal data were obtained from a third party (i.e., data not owned or collected by the authors), please explain how others can access or request the specific datasets related to your research. Confirm that others would be able to access or request these data in the same manner as the authors. Please also confirm that the authors did not have any special access or request privileges that others would not have.

- Minimal data were not obtained from a third party.

c) If there are no restrictions, and your minimal data were not obtained from a third party, please upload the minimal anonymized data set necessary to replicate your study findings to a stable, public repository and provide us with the relevant URLs, DOIs, or accession numbers. Please see http://www.bmj.com/content/340/bmj.c181.long for guidelines on how to de-identify and prepare clinical data for publication. For a list of recommended repositories, please see https://journals.plos.org/globalpublichealth/s/recommended-repositories. You also have the option of uploading the data as Supporting Information files, but we would recommend depositing data directly to a data repository if possible.

- There are no legal or ethical restrictions on the sharing of the minimal data set used in our analysis. The data have been anonymized and prepared in accordance to PLOS de identification standards.
- We have uploaded the dataset as a supporting information file labelled “REVISIONS MANUSCRIPT FOR COVID 19 VACCINE DATA.dta” and “COVID-19_vaccine_KAA_data.CSV”.
