# Supplementary material for: Knowledge, attitudes and acceptance of COVID-19 vaccine among pregnant women in Mbeya Region
Source: PLOS Glob Public Health. 2025 Jul 24;5(7):e0004408. doi: 10.1371/journal.pgph.0004408 (PMC12288997; doi:10.1371/journal.pgph.0004408)
Supplement: Attachment — Submitted filename: Response to Reviewers_015654.docx [file pgph.0004408.s006.docx]

Can you provide a citation for: "The COVID-19 pandemic impacted numerous countries globally, including those in Africa? Africa was the last continent affected by the pandemic and was anticipated to be the region where the disease would spread rapidly and exert significant impact." In particular - *who* anticipated it to be the region with the most impact?

- We have provided citation to reflect that Africa was the last continent affected by the COVID-19 pandemic and was anticipated by global health authorities and researchers to face severe impacts due to fragile health systems and limited resources .

Can you move study limitations to the discussion prior to the conclusion?

- Yes, we have relocated limitations sections to the end of discussion, just before the conclusion to align with standard practice in most journals.

I think you can have either the specific objectives or the research questions - both seem redundant.

- You are right to note that both may be redundant, we have used specific objectives and removed research questions.

I would delete Figure 1- I don't think pie charts provide much information.

- Action : Deleted figure 1

For Figure 2 - could you re-order form highest to lowest?

- Action: Re-ordered figure 2 from highest to lowest.

Results - when you mention age in the paragraph, can you put in "years" as well?

- Action : Revised to 30 years

Note in terminology - "peasant" in North American English can seem to be derogatory. You are free to keep that, especially if that is the term people use to self-identify, but "farmer" or the like seems potentially more interpretable (?)

- You are right “peasant” can sound derogative especially in North American English, we have replaced it with “farmer”.

In the intro, you have "A study conducted in Tanzania indicated that COVID-19 vaccine hesitancy was approximately 65%" - what did they mean by 65% vaccine hesitant? Was that how many people refused a vaccine, or were questioning it? Different studies have different definitions of vaccine hesitancy, so can be good to clarify.

- A study conducted in Tanzania indicated that approximately 65% of the people were reluctant to get vaccinated against COVID-19.

I do not think Figure 3 adds much. I only include Figures if they include more information beyond what you can say in a paragraph text. I could maybe think of somehow including a bar chart of the different knowledge questions, and how often people responded correctly to them?

- Figure 3 summarizes the results extracted from bloom cut off points to grade the level of knowledge on COVID-19 vaccine; The response to knowledge questions are summarized in the table 3.

Figures 4-6 also don't add much to the article.

- Likewise figure 4 summarizes the results extracted from bloom cut off points to grade the attitude patterns towards COVID-19 vaccination; The response to attitude questions are summarized in the table 4.
- Figure 5 marks a repetition to a response question in table 4, so it has been removed.

I would personally prefer you to write out KAA throughout the manuscript instead of relying on an acronym.

- We have spelled out “Knowledge, attitude and acceptance” instead of using” KAA” especially for clarity among readers.

In the limitations in discussion, I would make that narrative instead of using bullet points.

- We have reformatted the limitations as narrative text instead of bullets points.
